# Hemophagocytic Lymphohistiocytosis (HLH) Following Immune Checkpoint Therapy (ICT)

**DOI:** 10.1155/crom/5582848

**Published:** 2025-01-06

**Authors:** Saivaroon Gajagowni, Emily Wang, Jianbo Wang, Matthew T. Campbell, Bilal A. Siddiqui

**Affiliations:** ^1^Division of Internal Medicine, Baylor College of Medicine, Houston, Texas, USA; ^2^Division of Hospital Medicine, The University of Texas MD Anderson Cancer Center, Houston, Texas, USA; ^3^Pharmacy Clinical Programs, The University of Texas MD Anderson Cancer Center, Houston, Texas, USA; ^4^Department of Genitourinary Medical Oncology, The University of Texas MD Anderson Cancer Center, Houston, Texas, USA

**Keywords:** hemophagocytic lymphohistiocytosis, immune checkpoint therapy, renal cell carcinoma

## Abstract

In the past decade, the use of immune checkpoint therapy (ICT) has increased across many malignancies, including metastatic renal cell carcinoma as an option for frontline and subsequent lines of therapy. Despite the many therapeutic benefits of ICT, its use is complicated by the potential risk of immune-related adverse events (irAEs). One rare but potentially life-threatening irAE is hemophagocytic lymphohistiocytosis (HLH). HLH is a systemic inflammatory disorder resulting in multiorgan failure. The diagnosis of HLH is a challenge due to nonspecific symptoms and overlap with other systemic conditions, which can lead to delays in receiving appropriate treatment and potentially poor patient outcomes. This case illustrates the management of HLH caused by nivolumab plus ipilimumab combination therapy through the use of corticosteroids and tocilizumab in a patient with metastatic clear cell renal cell carcinoma.

## 1. Introduction

T cells play an essential role in antitumor immune activity. Activation and proliferation of T cells are reliant on the binding of the T cell receptor with the major histocompatibility complex and peptide on antigen-presenting cells (APCs) and the binding of CD28 on T cells to the costimulatory B7 ligand on APCs. Inhibitory immune checkpoints, such as cytotoxic T lymphocyte–associated protein 4 (CTLA-4) and programmed death 1 (PD-1), are upregulated to inhibit unrestrained T cell activation. Immune checkpoint therapy (ICT) has been developed to block these inhibitory molecules and increase T cell-mediated antitumor activity [1]. The use of immunotherapy has increased in recent years as more patients are eligible for ICT treatment [2].

Despite their potential, the use of ICT is complicated by immune-related adverse events (irAEs). The incidence of irAEs ranges from 15% to 90% depending on which agent is used and the length of treatment [3]. Loss of self-tolerance, cross-reactivity between tumor and healthy tissue, and a proinflammatory state caused by increased inflammatory cytokine production are all mechanisms implicated in the pathophysiology behind irAEs [4]. As a result, irAEs can affect almost any organ and range in severity of toxicity. Hemophagocytic lymphohistiocytosis (HLH) is a systemic inflammatory disorder characterized by aberrant activation of natural killer cells, cytotoxic T cells, and macrophages with resulting multiorgan failure and coagulopathy [5]. Primary HLH is characterized by inborn errors of immunity leading to immune overstimulation. Secondary HLH occurs in the absence of a heritable defect and is induced by underlying triggers such as infection and malignancies. Overall mortality from HLH ranges from 30% to 40%, with rates as high as 60% being reported in the setting of primary HLH [6, 7]. The increased mortality is partly due to the paucity of diagnostic tools and the overlap of symptomology with other conditions such as infection or malignancy, leading to delayed diagnosis and initiation of treatment [8]. It is therefore important to understand the clinical presentation and diagnosis of HLH secondary to ICT. Due to the rarity of the condition, only a few cases exist [9]. We present a case report of ICT-induced HLH in a patient treated with nivolumab plus ipilimumab for metastatic renal cell carcinoma (RCC) with a focus on diagnostic and therapeutic interventions.

## 2. Case Presentation

A 63-year-old female with a past medical history of hypertension, hypothyroidism, psoriatic arthritis, and metastatic clear cell RCC (intermediate risk per scoring guidelines) involving bilateral kidneys, bone, and lymph nodes was initiated on first-line nivolumab plus ipilimumab combination therapy. The patient had received three full cycles of nivolumab plus ipilimumab combination therapy, with the last cycle approximately 2 weeks prior to presenting to the emergency center with a 1-day history of new-onset fever, fatigue, and nausea.

Upon arrival, the patient was hypotensive and febrile to 39.5°C. Laboratory results revealed pancytopenia with white blood cell (WBC) 3.8 K/*μ*L (range 5.1–10.5 K/*μ*L), hemoglobin (HgB) 10.3 g/dL (range 12.2–15.3 g/dL), platelets (plts) 52 K/*μ*L (range 160–397 K/*μ*L), and absolute neutrophil count (ANC) 2.62 K/*μ*L (1.95–7.25 K/*μ*L). The patient was admitted and started on broad-spectrum antibiotics while an infectious workup was ordered. This workup was negative but the patient continued to have fevers, so a broad workup was ordered. Workup revealed ferritin of 20,405 ng/mL (range 13–150 ng/mL), triglyceride (TG) 416 mg/dL (range < / = 149 mg/dL), CRP 158.99 mg/L (range < / = 10 mg/L), LDH 745 U/L (range 135–214 U/L), fibrinogen 320 mg/dL (range 214–503 mg/dL), IL-6 153 pg/mL (range < / = 5 pg/mL), IFN*γ* 220.2 pg/mL (range < / = 5 pg/mL), TNF-*α* 220.2 pg/mL (range < / = 22 pg/mL), and IL-2 soluble receptor of 18,359.5 pg/mL (range 175.3–858.2 pg/mL). Hematology and leukemia teams were consulted for further management. A bone marrow biopsy was completed to evaluate her pancytopenia and showed occasional phagocytic histiocytes.

**Figure 1:**
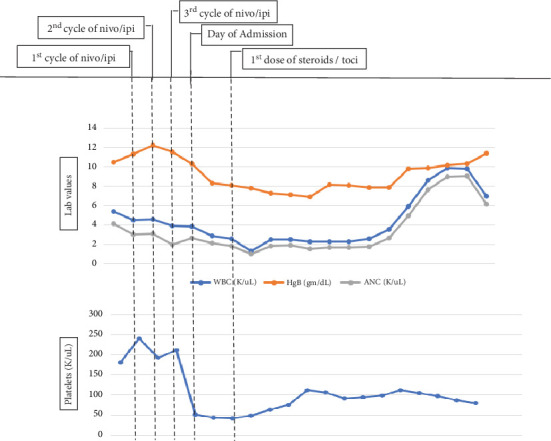
Hematologic trends from the initiation of ICT through duration of treatment for ICT-induced HLH. Abbreviations: g/dL = grams per deciliter, HLH = hemophagocytic lymphohistiocytosis (HLH), ICT = immune checkpoint therapy, ipi = ipilimumab, K/*μ*L = thousands per cubic milliliter, nivo = nivolumab, toci = tocilizumab.

**Figure 2:**
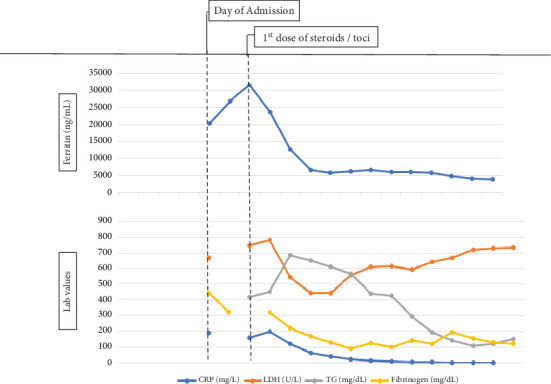
HLH laboratory trends from day of admissions through duration of treatment for ICT-induced HLH. Abbreviations: CRP = c-reactive protein, LDH = lactate dehydrogenase, ng/mL = nanograms per milliliter, TG = triglycerides, toci = tocilizumab.

Given these findings, treatment with dexamethasone 20 mg intravenous (IV) daily was initiated, along with two doses of tocilizumab 8 mg/kg provided within a 24-h time frame. Immediately, the patient defervesced with the last fever documented prior to receiving dexamethasone and tocilizumab. After 6 days of dexamethasone 20 mg IV daily, the patient's plts had improved to 100 K/*μ*L with stable WBC 2.3 K/*μ*L (range 4.1–10.5 K/*μ*L) and ANC 1.65 K/*μ*L (range 1.95–7.25 K/*μ*L) (Figure 1). Additionally, ferritin levels trended down from a peak of 31,477 ng/mL after the first dose of dexamethasone to 3908 ng/mL upon discharge (Figure 2). Other HLH laboratory values such as fibrinogen, CRP, and TG decreased as well after the initiation of steroids. The cytokines IL-6, IFN*γ*, and TNF-*α* fluctuated during admission; however, they were notably undetectable 6 days after initiating steroids and 4 days after having received two doses of tocilizumab.

The patient was discharged on steroids alone with plan for outpatient tapering. At the most recent follow-up, the patient remains on dexamethasone 1 mg PO daily with WBC 4 K/*μ*L (range 4.1–10.5 K/*μ*L), HgB 8.6 g/dL (range 12.2–15.3 g/dL), plts 225 K/*μ*L (range 160–397 K/*μ*L), and ferritin 472 ng/mL (range 13–150 ng/mL). She has not required any additional treatment for her HLH and remains free from any packed red blood cells (pRBC) or platelet (plt) transfusions. From a metastatic RCC perspective, she was followed with imaging for 5 months without resumption of ICT. She experienced disease progression at that time with multiple growing bilateral masses in her kidneys. She was started on axitinib as a second-line therapy with the decision to not rechallenge with ICT. On her initial restaging on axitinib, she had a reduction in the size of multiple bilateral renal masses consistent with favorable disease response.

## 3. Discussion

We present a case of HLH due to nivolumab plus ipilimumab combination therapy in a patient with RCC. We add to the limited existing database of ICT-induced HLH by presenting only the second reported case in a patient with RCC treated with nivolumab plus ipilimumab. Further, our case is unique in that it includes targeted therapy for HLH with tocilizumab.

The pathophysiology of HLH in immunotherapy is thought to be secondary to aberrant T cell activation due to the downregulation of inhibitory signals [10]. Further, PD-1 expression by tumor-associated macrophages inhibits phagocytosis and the use of PD-1 inhibitors has been shown to reverse this effect [11]. In our patient, the use of ipilimumab to inhibit CTLA-4 and nivolumab to inhibit PD-1 led to both increased T cell activation and macrophage phagocytosis which are the hallmarks of HLH. Specific risk factors for developing HLH secondary to immunotherapy are unknown. Existing studies suggest a genetic predisposition to the condition if there are mutations in genes associated with primary HLH [5].

Diagnosis of HLH remains a challenge due to nonspecific symptoms and overlap with other systemic conditions. Specific laboratory values to monitor include elevated ferritin (usually greater than two times the upper limit of normal), hepatic transaminases, and lactate dehydrogenase with hypofibrinogenemia and cytopenia. Clinical manifestations include fever, acute kidney injury, neurotoxicity, or pulmonary symptoms [5]. Recently, the soluble IL-2 receptor has gained popularity as a low-cost diagnostic test for HLH with a higher sensitivity than ferritin, with an area under the curve of 0.90 (95% confidence interval, 0.83–0.97) compared with an area under the curve of 0.78 (95% confidence interval, 0.67–0.88) for ferritin [12, 13].

It is important to distinguish HLH from cytokine release syndrome (CRS) and immune effector cell–associated hemophagocytic lymphohistiocytosis-like syndrome (IEC-HS). The inflammatory state seen in CRS is mediated by cytokines such as IL-6 and is often seen after chimeric antigen receptor (CAR)-T cell therapy or with therapeutic monoclonal antibody infusions. Differentiating CRS from HLH can be difficult due to overlapping lab values, but biopsy does not show hemophagocytosis in CRS. This distinction is important because treatment for HLH involves immunosuppression while CRS involves cytokine blockade, especially against IL-6 [14]. IEC-HS is defined as pathologic hyperinflammatory syndrome that (1) manifests with features of macrophage activation/HLH (e.g., hemophagocytosis); (2) is attributable to IEC therapy; and (3) is associated with progression or new onset of cytopenias, hyperferritinemia, coagulopathy, and/or transaminitis [15]. This is primarily seen after CAR-T cell therapy where effector cells are externally modified. Although ICT can be considered IEC therapy because of the similar increase in T cell activity, it is driven by an endogenous process and is therefore distinct. The treatment for both, however, remains the same because of the shared pathophysiology.

As discussed above, treatment for HLH is aimed at addressing the excess immune activation via immunosuppression. Guidelines from HLH-2004 recommend initiation of corticosteroids and etoposide. Most patients see resolution with corticosteroid monotherapy, with only severe or refractory cases requiring initiation of chemotherapy. Uniquely, our patient was treated with tocilizumab for 2 days after the initial diagnosis. Tocilizumab is an anti–IL-6 receptor monoclonal antibody shown to be effective in the treatment of CRS in CAR-T therapy [16]. Clinical trials have used tocilizumab before administration of CAR-T or T cell bispecific antibodies and have shown reduced rates of CRS without significant adverse effects [17, 18]. Although IL-6 is more commonly associated with CRS, the elevation seen in our HLH patient suggested a possible therapeutic target similar to that seen in CRS. This shared mechanism is the rationale for using tocilizumab to treat HLH associated with immunotherapy.

The use of this medication in HLH represents a shift toward targeted therapy via inhibition of cytokines and signaling molecules implicated in the inflammatory pathway [19]. Clinical trials using therapies targeting IFN*γ*, janus kinase (JAK), CD52, IL-1, IL-6, and IL-18 are ongoing and showing promising results. Early use of anakinra (IL-1 receptor antagonist) in pediatric patients with secondary HLH resulted in decreased mortality and a 57% reduction in serum ferritin levels [20]. In our patient, treatment with dexamethasone and tocilizumab brought down her ferritin by 50% within 3 days, supporting the use of this therapy in HLH secondary to immunotherapy with elevated IL-6. Further clinical trials are needed comparing the use of tocilizumab to other agents both as prophylaxis and therapy to determine its role in HLH. Lastly, we decided not to restart our patient on immunotherapy postrecovery from HLH. It is important to note that our patient responded to immunotherapy and that irAEs, although serious, can be suggestive of antitumor efficacy and have been associated with improved survival outcomes [21]. However, improved survival must be balanced with the disability caused by irAEs. One way to distinguish between irAEs is via grading from 1 to 4 based on severity and management [22]. Grade 3–4 irAEs require hospital/ICU admission and are treated with high-dose steroids. Our patient's irAE is characterized as Grade 3 due to requiring hospital admission and initiation of steroids/tocilizumab. Rechallenging for Grade 3 irAEs is rare. Despite the possible efficacy associated with treatment, we decided not to rechallenge our patient due to the risk of recurrence of HLH.

In conclusion, we presented a case of HLH secondary to treatment with nivolumab plus ipilimumab for RCC. This case is unique in the use of targeted therapy with tocilizumab for the treatment of HLH. This case reviewed the important diagnostic principles and advancements in the management of HLH, especially in the setting of immunotherapy.

## Data Availability

The data that support the findings of this study are available from the corresponding author upon reasonable request.
